# The ocular surface bacterial contamination and its management in the prophylaxis of post cataract surgery endophthalmitis

**DOI:** 10.22336/rjo.2021.2

**Published:** 2021

**Authors:** Simina Daniela Soare, Larisa Ilie, Otilia Costeliu, Ana Cristina Ghiță, Liliana Mary Voinea, Aurelian Mihai Ghiță

**Affiliations:** * Lifecare Eye Clinic, Buftea, Romania; ** Department of Ophthalmology, University Emergency Hospital, Bucharest, Romania; *** Ocularcare Eye Clinic, Bucharest, Romania; **** Department of Physiology II, “Carol Davila” University of Medicine and Pharmacy, Bucharest, Romania

**Keywords:** ocular surface, bacterial contamination, prophylaxis of post-cataract surgery endophthalmitis, cataract surgery, bacterial spectrum

## Abstract

**Objective:** To investigate the recent pieces of evidence regarding the bacterial ocular surface contamination and its treatment in the prophylaxis of post-cataract surgery endophthalmitis.

**Methods:** We conducted a literature research on the topic of interest and selected the most relevant data.

**Results:** The studies reported a relatively high rate of positive conjunctival culture and the most frequently isolated organism was Coagulase negative Staphylococcus, which is also the most common etiological agent of the postoperative endophthalmitis. The bacterial ocular surface load is influenced by age, climate, associated diseases, topical and systemic medication. The use of povidone-iodine alone or in association with levofloxacin eyedrops as prophylactic method is effective in reducing the conjunctival bacterial contamination and consequently decreases the incidence of postoperative endophthalmitis.

**Conclusions:** Based on the current pieces of evidence, adequate treatment of the bacterial ocular surface contamination prior to cataract surgery seems to be effective in preventing endophthalmitis after cataract surgery.

**Abbreviations:** EU = European Union, Spp. = Species, HIV = Human Immunodeficiency Virus

## Introduction

Cataract is the leading cause of world blindness, affecting almost 18 million people worldwide [**1**]. It is responsible for 47.8% to 51% of all global blindness [**2**]. The cataract treatment is relatively easy, safe and cost-efficient and due to the latest advances in the technique and instruments used, it has become the most frequently performed surgical procedure in the world [**3**]. 4.7 million cataract surgeries were performed in the European Union (EU) in 2017, out of which 77,456 in Romanian hospitals [**4**]. In 2017, in Romania, 395,44 cataract surgeries were conducted per 100.000 inhabitants, ranking Romania on the 23rd position in the EU [**4**].

Cataract surgery is one of the safest surgical procedures with a complication rate ranging between 1.7% and 2.0%. Whilst, most of the periprocedural complications are easy to manage, endophthalmitis, although rare, remains one of the most severe complications leading to high visual morbidity despite appropriate treatment [**5**-**7**]. According to different studies, postoperative endophthalmitis incidence varies from 0,04 to 0,2% [**8**-**11**]. The factors known to be associated with endophthalmitis can be divided into two categories: patient-related factors (ocular tear film, ocular adnexa, comorbidities, age, climate, sex and the use of alpha agonists) and factors related to the surgical procedure (irrigation solutions, surgical instruments, respiratory and skin flora of the surgeon and nurses, operating theatre air, surgeons’ experience) [**6**,**12**].

The main source of bacteria in postoperative endophthalmitis is the patient’s conjunctival flora, which enters in the anterior chamber during the surgical procedure, through incisions. However, the contamination of surgical equipment such as instruments, supplies, prepared solutions, surgical fields, artificial intraocular lenses, should always be taken into consideration [**6**,**13**,**14**]. 

The etiology of 60% to 80% of postoperative endophthalmitis is represented by bacteria found in the eyelid margin and tear film [**6**]. Among the isolated pathogens, Staphylococcus epidermidis (coagulase-negative) is the most frequently identified (50-85%), followed by Staphylococcus aureus and Streptococcus Spp. (Species). 20% of the infections are caused by gram negative microorganisms [**15**]. 

Considering the information outlined above, it should be mandatory to analyze the conjunctival swabs and treat any conjunctival contamination prior to cataract surgery. 

## Methods

We reviewed the literature and summarized the findings on bacterial conjunctival contamination and its treatment prior to cataract surgery, in order to reduce the incidence of postoperative endophthalmitis.

## Results

Group of risk for high bacterial contamination

Contamination of the conjunctiva is a fact but, the environmental factors, comorbidities and ocular surface changes modified the incidence of bacterial conjunctival contamination and the spectrum. From literature, we highlighted the external and internal conditions associated with an increase of the rate of bacterial contamination.

Climate

It appears that conjunctival bacterial contamination of patients undergoing cataract surgery presents a seasonal prevalence pattern and this can be considered a risk factor for developing postoperative endophthalmitis during certain months of the year [**16**,**17**]. This was stated for the first time by Smith C.H. in 1954 and supported by Rubio, who conducted a study on 4.432 patients, who showed an increased frequency of the positive bacterial results during April, May, and June. Furthermore, Streptococcus pneumoniae had an increased isolation rate in March, November and December, while Haemophilus Spp. had higher isolation rates in January and April [**16**,**18**].

Age

The reviewed studies found that the conjunctival bacterial contamination increases with age, due to a reduction in the local and systemic immunity and to a higher incidence of associated diseases [**19**]. In a sample of 794 patients, Rubio E.F. found that patients older than 64 years had more conjunctival flora and more conjunctival density than those younger than 65 years [**20**]. On a larger sample of 19.426 patients, Norregaard et al. showed that advanced age increases the risk of postoperative endophthalmitis due to an increase of the conjunctival bacterial contamination [**21**]. In a retrospective case-control study on 22.091 enrolled patients, Montan et al. described a trend towards a higher risk of postoperative endophthalmitis in patients older than 81 years [**22**]. In a study comprising 579 subjects, Chikako et al. showed that patients older than 60 years had a statistically significant higher bacterial contamination rate than the ones younger than 60 years (p<0.001) [**19**]. Also, Kawata showed that the positive bacterial culture was related with older age (p<0,001) [**23**]. The bacterial detection rate according to age was 16.4% in patients younger than 60 years, 40.7% in patients aged between 61 and 70 years, 39.9% in patients aged between 71 and 80 years and 51.5% in patients older than 81 years [**19**]. 

Diabetes mellitus

The conjunctival flora pattern of diabetic patients is characterized by an increased number of bacteria, which is a predominant cause of many diabetic infections including postoperative endophthalmitis. Several studies on the conjunctival bacterial contamination of diabetic patients were published and showed contradictory results regarding the correlation between diabetes mellitus and positive bacterial cultures. In a study including 590 patients, Kawata et al. showed that patients with diabetes mellitus have a higher rate of positive bacterial culture [**23**]. De Kaspar found that patients with systemic risk factors, including diabetes mellitus, have a higher risk of positive bacterial culture [**24**]. These results were supported by further studies showing a higher incidence of positive bacterial culture prior to cataract surgery [**23**,**25**,**26**]. However, Chikako et al. showed that there was no statistically significant difference between diabetic and non-diabetic patients [**19**]. Another study found that diabetic patients are less likely to be colonized by methicillin-resistant organisms (p<0.02) [**27**]. Arbab et al. supported this result after studying 80 patients [**28**].

In a study of 5.922 patients, out of whom 1.325 were diabetics, conducted by Rubio et al., diabetic patients had a higher prevalence of positive conjunctival cultures for Staphylococcus aureus, Enterococcus, Streptococcus and Klebsiella spp. [**29**]. This study also assessed the conjunctival contamination in relation to the creatinine serum levels. 9.27% of the patients with suspected kidney disease had a higher rate of positive conjunctival bacterial culture [**30**,**31**].

Bilen et al. showed an increased prevalence for Staphylococcus aureus only in patients with type 2 diabetes, but the number of the studied patients was lower than in the study cited above [**32**]. Martins et al. studied only the patients with diabetic retinopathy and found only an increased prevalence of Staphylococcus coagulase-negative [**26**]. However, this final study used certain exclusion criteria for their patients, such as external ocular infection or inflammation, which were not used in the study conducted by Rubio. In addition, their patients were younger (mean age was 67 years old), which could have influenced their conjunctival flora [**26**].

Arbab et al. conducted a study on 80 patients concluding that the presence of diabetic retinopathy increased the risk of positive conjunctival cultures among diabetic patients [**28**]. 

Moreover, patients with diabetic retinopathy have a higher likelihood of having >2 isolated bacteria from the conjunctival culture than patients without retinopathy [**28**].

HIV infection

HIV (Human Immunodeficiency Virus) infection is characterized by a decreased immune response that leads to a higher rate of positive conjunctival cultures. Giles et al. conducted a study on a sample of 104 HIV patients out of whom 62.50% had positive conjunctival cultures [**33**]. The study also showed that the conjunctival bacterial load was significantly correlated with the duration of antiretroviral therapy and the degree of immunosuppression. Fontes et al. found similar results (67%) regarding the rate of positive conjunctival cultures, but showed no correlation between the level of immunosuppression and ocular flora [**34**]. Gritz et al. found that the conjunctival flora is similar in HIV negative and positive patients [**35**]. The identified pathogens were similar to those identified by other studies conducted on people without HIV infection: Coagulase-negative Staphylococcus (90,77%) and Staphylococcus aureus (4,61%) [**33**,**35**]. However, preventive measures should be taken in HIV patients prior to cataract surgery in order to reduce the risk of developing postoperative endophthalmitis.

Other factors

Other factors that may influence the conjunctival bacterial contamination are hyperlipidemia, dry eye syndrome and the use of topical ocular medication [**36**]. Chikako et al. conducted a study that comprised 579 patients who underwent cataract surgery and showed that the factors listed above were associated with significantly lower bacterial detection rates [**19**]. It is possible that the conjunctival sac and its bacterial flora, which are located upstream of the nasolacrimal duct, might be affected by the changes in the composition of the nasolacrimal duct fluid secondary to hypercholesterolemia. Honda et al. compared bacterial isolation rates from the conjunctival sac between patients receiving topical medication for glaucoma and those not receiving such medication. The bacterial detection rate in the topical medication group was significantly lower than the rate in the group without topical treatment and thus, a washout effect of the eye drops might be involved [**37**].

Moreover, another prospective study made in Japan found that bacterial isolation rates were significantly lower in patients suffering from dry eye syndrome and actively using eye drops compared to those that did not [**37**].

Other investigated factors such as HIV, immunosuppression, pregnancy and reproductive status (postmenopausal women versus women of reproductive age) do not interfere with the composition of conjunctival bacterial flora [**35**].

Incidence of positive cultures

The reviewed studies found a bacterial conjunctival positivity rate ranging between 12.2% and 92.85% [**19**,**38**-**44**] (**Table 1**). The differences may arise from the fact that the studies were conducted in different regions of the world that are at different levels of development. Only 2 studies were conducted in the same country (India), but the results were contradictory, one study showing a 12.2% positivity rate whilst the other one a 64% positivity rate [**41**,**44**]. The difference may arise from the fact that Gautam et al. included in the study patients undergoing both cataract and refractive surgery thus, the mean age was younger than the one from Sharma et al., who enrolled only patients undergoing cataract surgery. As previously mentioned, studies showed that an older age leads to a higher rate of positive conjunctival cultures.

**Tabel 1:** Incidence of positive cultures

Author, date	Country	Number of eyes included	Positive	Negative
Chikako 2012 [**19**]	Japan	579	39.2%	60.8%
Walker 1986 [**38**]	United Kingdom	100	74%	26%
Cham 2010 [**39**]	Philippines	30	90%	10%
Keshav 2012 [**40**]	Oman	112	48.3%	51.7%
Gautam 2017 [**41**]	India	500	12.2%	87.8%
Aghi 2016 [**42**]	Iraq	334	92.85%	7.15%
Reza 2008 [**43**]	Pakistan	170	52.4%	47.6%
Sharma 2013 [**44**]	India	200	64%	36%
De Kaspar 2009 [**24**]	Germany	1474	14.9%	85.1%

Identified bacteria

The main source of bacteria causing postoperative endophthalmitis is represented by the indigenous bacteria from the ocular surface [**10**]. Dong et al. conducted a study that analyses the microbial diversity of the ocular surface by using DNA (Deoxyribonucleic Acid) sequencing-based detection and identification of bacteria. The study found that 96% of the conjunctival microbiota was represented by the following 12 genera: Pseudomonas, Propionibacterium, Bradyrhizobium, Corynebacterium, Acinetobacter, Brevundimonas, Staphylococci, Aquabacterium, Sphingomonas, Streptococcus, Streptophyta and Methylobacterium [**47**]. Coagulase-negative Staphylococcus was the most frequently identified bacteria in the conjunctival flora in all the reviewed studies, being isolated in 18.0% to 89% of the conjunctival cultures, making it the most common conjunctival bacteria worldwide. Staphylococcus aureus was identified in 1.8%-72.31% of the conjunctival cultures. Corynebacterium spp. was isolated in 1%-46.79% of the conjunctival cultures. Propionibacterium spp. was isolated in 5.4%-33.00% of the conjunctival cultures. Less frequently isolated species were the following: Streptococcus spp. (1.8%-8%), Pseudomonas spp. (0,56%-2%), Proteus spp. (0.8%-2%), Enterococcus spp. (1.8%-8%) (**Table 2**). The disparities found between these studies can be explained by the fact that the studies were conducted in different countries, the number of patients included in the studies ranged between 30 and 5.922 and the mean age of patients varied [**19**,**24**,**37**-**42**,**44**-**46**]. 

**Tabel 2:** Identified bacteria among the studies

Study	Eyes	Staphyloccocus Epidermidis	Staphyloccocus Aureus	Enterococcus spp.	Bacillus spp.	Propionibacterium spp.	Streptococcus spp.	Pseudomonas spp.	Proteus spp.	Corynebacterium spp.
Rubio et al. 2009 [**45**]	5922	87.32%	9.73%	2.15%	0.63%	25.08%	3.24%	0.56%	1.71%	46.79%
De Kaspar 2009 [**24**]	1474	70,1%	3.8%	-	0.8%	5.4%	-	-	0.9%	14.4%
Cham et al. [**39**]	30	89%	12%	8%	45%	-	-	-	-	-
Faria and Arantes et al. [**46**]	50	54.0%	12.0%	2%	8.0%	-	-	-	2%	2%
Chikako et al. [**19**]	579	67.0%	3.9%	2.7%	5.9%	-	3.2%	2%	0.8%	26.7%
Walker et al. 1986 [**38**]	100	58%	7%	-	1%	-	-	1%	-	-
Ohtani 2017 [**37**]	68	41%	8%	-	-	33%	1%	-	-	4%
Kesahv 2012 [**40**]	112	81,5%	1,8%	1,8%	-	-	11,1%	-	1,8%	1,8%
Gautam 2017 [**41**]	500	18.0%	72.31%	-	-	-	2%	-	-	-
Aghi 2016 [**42**]	334	47,10%	5.16%	-	4,70%	-	1,94%	-	-	31,60%
Sharma 2013 [**44**]	200	34%	14%	-	5%	-	2%	-	-	1%

Microbial resistance

Numerous studies evaluated conjunctival bacterial flora and its antibiotic resistance pattern in eyes of the patients undergoing cataract surgery. Tiago Eugenio et al. conducted a retrospective study on 50 patients undergoing cataract surgery, from August to October 2004. Out of 50 eyes, 86% had positive cultures, the most frequent microorganism being coagulase-negative Staphylococcus, found in 54% eyes. The isolation of coagulase-negative Staphylococcus showed high susceptibility rate to vancomycin, chloramphenicol, ciprofloxacin, gentamicin, oxacillin, cefotaxime. Staphylococcus aureus was found in 8% of the eyes and showed a 100% susceptibility rate to the previously mentioned antibiotics [**46**].

Rubio et al. conducted a prospective observational study on 1.940 patients undergoing cataract surgery, over a 12 months period. Coagulase-negative Staphylococcus had a prevalence of 88.3% and showed a high susceptibility rate to vancomycin, gentamicin, ciprofloxacin, and chloramphenicol. Staphylococcus aureus was found in 10.2% of the eyes and was sensitive to gentamicin, vancomycin, chloramphenicol, ciprofloxacin, and oxacillin. These results were similar to those found in the previous study [**45**].

Kehav R. Belur et al. conducted a prospective study on 56 patients scheduled for cataract surgery. Coagulase-negative Staphylococcus was isolated in 81.5% of the eyes; maximum sensitivity was achieved with vancomycin, gentamicin, chloramphenicol, and ciprofloxacin (**Table 3**). Staphylococcus aureus was isolated in 1.8% of the eyes with a 57% susceptibility rate to oxacillin and low susceptibility rate to vancomycin, gentamicin, and ciprofloxacin. These results were similar to those found in the studies mentioned above except for the resistance pattern [**40**] (**Table 4**). 

**Tabel 3:** Sensitivity and resistance of Coagulase-negative Staphylococcus to antibiotics according to different literature studies

		Gentamicin	Cefotaxime	Oxacillin	Vancomycin	Chloramphenicol	Ciprofloxacin
Tiago Eugenio [**46**]	Sensitive	85.2%	85.2%	85.2%	100%	92.6%	88.9%
	Resistant	14.8%	7.4%	14.8%	0%	7.4%	7.4%
Rubio [**45**]	Sensitive	84.7%	51.2%	51.3%	100%	96.7%	74.3%
	Resistant	15.3%	48.8%	48.7%	0%	3.3%	25.7%
Keshav R. Belur [**40**]	Sensitive	87%	-	50%	98%	68%	84%
	Resistant	3%	-	50%	2%	32%	14%

**Tabel 4:** Sensitivity and resistance of Staphylococcus aureus to antibiotics in different studies from literature

		Gentamicin	Cefotaxime	Oxacillin	Vancomycin	Chloramphenicol	Ciprofloxacin
Tiago Eugenio [**46**]	Sensitive	100%	100%	100%	100%	100%	100%
	Resistant	0%	0%	0%	0%	0%	0%
Rubio [**45**]	Sensitive	94.9%	42.6%	86.2%	100%	98.5%	85.2%
	Resistant	5.1%	57.4%	13.8%	0%	1.5%	14.8%
Keshav R. Belur [**40**]	Sensitive	74%	-	43%	83%	63%	72%
	Resistant	26%	-	57%	17%	37%	27%

According to the study conducted by Rubio et al., Pseudomonas Aeruginosa was detected less frequently (0.41%) and had a high-rate susceptibility for ciprofloxacin, tobramycin, and imipenem. However, in the study conducted by Wolfgang Haas, Pseudomonas had a high rate of resistance for ciprofloxacin, tobramycin, and imipenem. These results may be contradictory due to the different number of patients included in the study: 1.940 patients for Rubio and 56 patients for Belur Kehav [**40**] (**Table 5**).

**Tabel 5:** Sensitivity and resistance of Pseudomonas Aeruginosa to antibiotics in different studies from literature

		Ciprofloxacin	Tobramycin	Imipenem	Polymyxin B	Ampicilin
Wolfgang Haas [**48**]	Sensitive	9.1%	4.5%	11.4%	4.5%	-
	Resistant	90.9%	95.5%	88.6%	95.5%	
Rubio [**45**]	Sensitive	96.4%	96.4%	98.8%	-	30.1%
	Resistant	3.6%	3.6%	1.2%	-	69.9%

According to the reviewed studies and medical practice, an increase in the bacterial resistance has been noticed due to the inappropriate and widespread use of antibiotics. The resistance microbial pattern is influenced by the following factors: age, level of care, ethnic group, environment, and antimicrobial prescribing policies used. Therefore, when treating a positive conjunctival culture, the ophthalmologists should take into account the above-mentioned factors and decide when to treat and to use judiciously the antibiotics in order to preserve their efficacy and reduce the microbial resistance [**48**-**50**].

Prophylaxis

Considering the high rate of positive conjunctival culture, preoperative treatment should be applied in order to sterilize the cultures and reduce the risk of postoperative endophthalmitis. The most common used prophylactic methods are povidone iodine and levofloxacin eyedrops. 

Povidone-iodine is a potent chemical antiseptic that can be used as a preoperative agent due to its minimal secondary effects and to its proven effect on reducing the conjunctival bacterial load prior to ocular surgery [**51**,**52**]. It is mainly used to wipe the eyelids, to flush the upper and lower conjunctival fornixes or it is given in eye drops. Its efficacity was studied since 1984 by Apt and Isenberg on a prospective study of 30 patients undergoing ocular surgery. The number of colonies and species in the povidone-iodine treated eyes decreased by 91%, respectively 50%, compared to the untreated control eyes, and these results were statistically significant [**53**]. These findings are confirmed by more recent studies showing the effectiveness of povidone-iodine in reducing the conjunctival bacterial load preoperatively. In 2012, in a prospective, randomized study on 263 eyes, Nentwich et al. showed that the use of 10% povidone-iodine drops reduces significantly the number of positive conjunctival cultures [**54**]. In 2015, Pettey et al. found that 10% povidone-iodine decreases the bacterial growth by 28% and the colony counts by 40% [**55**]. However, one study showed that low-concentration povidone iodine (5%) is less effective as the sole antiseptic agent [**56**]. Ta et al. found no difference between the effectiveness of 10% and 5% povidone-iodine in reducing bacterial load [**57**].

Levofloxacin is a third-generation fluoroquinolone that is used topically because it penetrates the cornea and achieves high intraocular concentrations. Bacteria have high rates of susceptibility to last generation fluoroquinolones compared to ciprofloxacin and ofloxacin [**52**]. In 2007, ESCRS Study showed a lower incidence of postoperative endophthalmitis in patients treated preoperatively with topical 0.5% levofloxacin compared to the placebo group (1.5/ 1000 patients versus 2.1/ 1000 patients) [**58**]. 

Another study showed that the bacterial load is decreased by 72.7% when using topical 0.5% levofloxacin. The addition of 5% povidone-iodine increases the bacterial elimination rate to 86.4%. Israel A.R. highlighted that the use of both povidone-iodine and topical levofloxacin is more effective in decreasing the conjunctival bacterial load compared to iodine alone [**59**]. 

## Conclusions

Cataract surgery is one of the most frequent surgical procedures performed in the world. Although it is considered as a safe procedure, with manageable complications, postoperative endophthalmitis still remains a sight threatening and challenging complication. Therefore, the most suitable way to avoid this disastrous issue is to prevent it, by adequately treating any preoperatively bacterial contamination known. 

A large proportion of conjunctival cultures prove to be positive and correlated with the following factors: age, climate, hyperlipidemia, diabetes mellitus, HIV infection, dry eye syndrome, and the use of topical ocular medication. Older age is associated with a higher risk of postoperative endophthalmitis due to an increased bacterial conjunctival load. The prevalence of the conjunctival bacteria varies according to the period of the year and could predetermine the seasonal incidence of endophthalmitis. Diabetic patients have a higher rate of positive conjunctival cultures, conjunctival bacterial load, sometimes with >2 identified bacteria that increases the risk of postoperative endophthalmitis. Additional factors that increase this risk are diabetic retinopathy and diabetic nephropathy, which are indicators of advanced diabetic disease. The conjunctival flora is similar in HIV positive and negative patients, but caution should be taken due to their immunosuppression status that increases the risk of infection. The chronic use of ophthalmic topical medication for diseases such as dry eye syndrome, glaucoma, and other ocular diseases, is associated with significantly lower bacterial rates that can be owed to a washout effect of the eye drops.

The main source of infection is represented by the bacterial flora located on the ocular surface. This is sustained by the fact that the most common pathogen identified globally and the most common causative agent of postoperative endophthalmitis is the Coagulase negative Staphylococcus. It has high susceptibility rate to vancomycin, chloramphenicol, ciprofloxacin, and gentamicin. The second most identified bacteria are Staphylococcus aureus with a high susceptibility rate to gentamicin, oxacillin, vancomycin, chloramphenicol, and ciprofloxacin. Isolated species of Streptococcus, Pseudomonas, Proteus, and Enterococcus were less frequently identified. The widespread use of antibiotics has led to an increase of bacterial resistance making it a serious public health issue that restrains the therapeutic options in front of a patient with positive conjunctival culture. Therefore, practitioners should address this problem by treating any positive conjunctival culture in accordance with the antibiogram result. 

It is of vital importance to reduce the risk of postoperative endophthalmitis by applying appropriate methods of prophylaxis. The goal of these prophylactic methods is to sterilize the conjunctival sac prior to intraocular surgery. The most common prophylactic treatment is povidone-iodine alone or in association with levofloxacin eyedrops. Their efficacy in reducing the incidence of postoperative endophthalmitis has been proven by their widespread use over the last 25 years. However, due to the increased antibiotic resistance pattern of conjunctival bacteria, future studies are needed to select the appropriate methods of prophylaxis and find new ones.

**Conflict of Interest**

The authors declare no conflict of interest.

**Acknowledgements**

None.

**Sources of Funding**

None.

**Disclosures**

None. 
